# Both perfusion pressure and flow are critical in abdominal normothermic regional perfusion!

**DOI:** 10.3389/ti.2026.16734

**Published:** 2026-07-16

**Authors:** Raphaël Giraud, Benjamin Assouline, Franz Immer, Yvan Gasche, Karim Bendjelid

**Affiliations:** 1 Intensive Care Division, Department of Acute Care Medicine, Geneva University Hospitals, Geneva, Switzerland; 2 Department of Anesthesiology, Pharmacology, Intensive Care and Emergency Medicine, Faculty of Medicine, University of Geneva, Geneva, Switzerland; 3 Geneva Hemodynamic Research Group, Faculty of Medicine, University of Geneva, Geneva, Switzerland; 4 Latin Program of Organ Donation, Geneva, Switzerland; 5 Swisstransplant, Bern, Switzerland; 6 Intensive Care Unit, Hirslanden Clinique des Grangettes, Chêne Bougeries, Switzerland

**Keywords:** abdominal normothermic regional perfusion, A-NRP, arterial pressure monitoring, cDCD, controlled donation after circulatory death

Dear Editors,

Abdominal normothermic regional perfusion (A-NRP) has emerged as a major advance in controlled donation after circulatory death (cDCD), improving organ utilization and post-transplant outcomes, especially in liver transplantation. Clinical series have shown lower rates of ischemic cholangiopathy and better graft outcomes when A-NRP is used instead of rapid recovery alone [1, 2]. Yet the hemodynamic monitoring of the reperfused abdominal compartment remains conceptually underdeveloped. In many settings, the adequacy of abdominal reperfusion is inferred mainly from extracorporeal pump flow and venous oxygen saturation sampled from the drainage line. We believe this is physiologically insufficient, because tissue perfusion depends on arterial pressure as much as on blood flow. In this regard, continuous arterial pressure monitoring of the reperfused compartment should be regarded as a critical requirement, because pressure, and not flow alone, makes organ perfusion effective [3, 4].

The rationale is grounded in fundamental hemodynamics. Organ blood flow is governed by the pressure gradient across the vascular bed and its resistance. Extracorporeal flow displayed by the pump is therefore only a circuit variable; it does not directly indicate whether an adequate driving pressure is being transmitted to the abdominal microcirculation. This issue is particularly relevant after death, when circulation is non-pulsatile and entirely pump-driven. Under these conditions, apparently acceptable circuit flow may coexist with inadequate arterial pressure if systemic vascular resistance is low. In such a situation, blood may circulate through the circuit while the pressure head required to perfuse ischemic tissues remains insufficient. In physiological terms, this corresponds to flow that is measurable, yet hemodynamically ineffective [4]. One may therefore wrongly assume that high blood flow necessarily implies adequate arterial pressure and tissue perfusion. Conversely, even apparently sufficient pressure may coexist with inadequate tissue oxygen delivery if cardiac output, or, here, circuit flow, is too low.

The kidney illustrates why this distinction matters. Renal perfusion becomes pressure-dependent below the autoregulatory range, and autoregulation itself is altered in shock states and cannot be assumed to be preserved after circulatory arrest and warm ischemia [4]. Thus, during A-NRP, low arterial pressure may translate directly into inadequate renal cortical perfusion despite apparently satisfactory extracorporeal flow. The same concern likely extends to the liver and splanchnic organs, whose recovery after warm ischemia depends on effective tissue oxygen delivery rather than simply on the presence of circulating blood in the aorta. Preserved macroflow is not synonymous with restored organ perfusion [4].

For the same reason, reliance on venous-line SvO_2_ is problematic. SvO_2_ is a global, flow-weighted average of oxygen extraction in the venous effluent reaching the sampling site. It is not an organ-specific perfusion marker. Low SvO_2_ is certainly concerning, but normal or elevated mixed SvO_2_ does not exclude regional hypoperfusion, heterogeneous flow distribution, impaired oxygen extraction, or microcirculatory shunting. Venous oxygen saturation can remain normal despite persistent tissue hypoxia, a phenomenon described as “covert tissue hypoxia” [5]. This is particularly relevant when perfusion is unevenly distributed, because well-perfused territories may dominate the venous signal and mask ischemia in more vulnerable organs.

As illustrated in Figure 1, extracorporeal pump flow does not necessarily translate into effective organ perfusion. While pump flow reflects circuit blood flow and venous-line SvO_2_ reflects a global mixed venous signal, arterial pressure measured within the reperfused abdominal compartment more directly reflects the driving pressure for abdominal organ perfusion.

**FIGURE 1:**
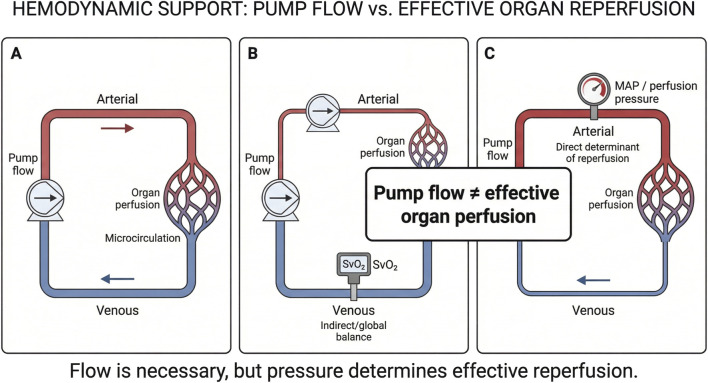
Conceptual framework for hemodynamic monitoring during abdominal normothermic regional perfusion (A-NRP). **(A)** Circuit overview. **(B)** Indirect monitoring. **(C)** Direct physiological target.

Evidence from extracorporeal circulation supports this concern. During cardiopulmonary bypass, mixed venous oxygen saturation has been shown to correlate poorly with regional venous saturations. McDaniel and colleagues demonstrated that normal mixed venous saturation did not exclude marked regional venous desaturation during bypass [6]. Dahn et al. showed that global venous oxygenation may coexist with depressed splanchnic venous oxygen saturation, indicating that visceral hypoxia may be missed when only systemic venous oxygenation is monitored [7]. Although these studies were not performed in A-NRP donors, their physiological message is directly transferable: global venous oxygenation is an indirect and potentially misleading surrogate for abdominal organ reperfusion.

Pump flow suffers from a similar limitation. It quantifies what the circuit delivers, not what the organ bed receives. If arterial pressure is low because resistance is low, a seemingly adequate flow can still fail to generate sufficient transorgan perfusion pressure. This is why coherence between macrocirculation and microcirculation cannot be assumed. In critically ill states, correction of global hemodynamic variables does not reliably restore microvascular perfusion, and the same principle likely applies to organs undergoing reperfusion after warm ischemia [8]. This blind spot may be even more pronounced in uncontrolled DCD, where longer no-flow and low-flow periods, more severe metabolic derangement, and greater vascular dysfunction are expected at the time NRP is initiated. Uncontrolled DCD donors undergoing NRP have been shown to display more severe metabolic derangement than controlled DCD donors, often requiring more aggressive corrective treatment during reperfusion [9]. A-NRP is intended to recondition organs, not merely to re-establish circuit circulation. Even at constant pump flow, a fall in vascular resistance in one abdominal territory may redistribute flow toward that organ at the expense of others. In this setting, total circuit flow alone cannot guarantee adequate and balanced reperfusion, because changes in regional resistance directly affect flow distribution and tissue perfusion.

Continuous arterial pressure monitoring of the reperfused abdominal compartment addresses this blind spot. It provides direct information on the driving force available to perfuse the kidneys, liver, and gut and allows real-time adjustment of ECMO flow and vasopressors. In practical terms, this pressure should ideally be measured directly within the reperfused abdominal arterial compartment, for example, through a side port connected to the arterial return line or through an arterial catheter placed in the infradiaphragmatic arterial circulation. This approach is also consistent with broader NRP standardization efforts, which emphasize hemodynamic control and procedural reproducibility during A-NRP [3]. Published A-NRP protocols support maintaining a mean arterial pressure in the range of 60–65 mmHg to ensure effective abdominal organ perfusion [3, 10]. The principle nevertheless remains straightforward: if arterial pressure is not monitored within the reperfused compartment, one cannot be confident that all abdominal organs are being effectively and adequately reperfused. This does not diminish the importance of adequate extracorporeal flow; rather, it emphasizes that flow must be interpreted together with pressure.

We are not claiming that compartmental arterial pressure monitoring has already been proven, in isolation, to improve graft or recipient outcomes. Rather, we argue that it is physiologically indispensable if the objective of A-NRP is true organ reperfusion rather than simple extracorporeal circulation. Flow is necessary, but pressure is what makes flow biologically relevant and appropriately distributed. In A-NRP, direct monitoring of arterial pressure within the reperfused compartment should therefore be considered a logical next step toward more rational, physiology-guided donor management.

## Data Availability

The original contributions presented in the study are included in the article/supplementary material, further inquiries can be directed to the corresponding author.
